# Feasibility of a prospective, longitudinal study of resilience among young military recruits with embedded laboratory sub-study: the ARMOR pilot trial

**DOI:** 10.21203/rs.3.rs-3112652/v1

**Published:** 2023-07-03

**Authors:** Andrea C Hitz, Shelly Bachelors Degree Hubbling, Annika Hodges, Emily M Hagel Campbell, Ann Bangerter, Melissa A. Polusny

**Affiliations:** University of Minnesota Medical School Twin Cities Campus: University of Minnesota Twin Cities School of Medicine; University of Minnesota Twin Cities College of Biological Sciences; Minneapolis VA Health Care System: Minneapolis VA Medical Center; Minneapolis VAHCS: Minneapolis VA Medical Center; Minneapolis VAHCS: Minneapolis VA Medical Center; Minneapolis VA Health Care System: Minneapolis VA Medical Center

## Abstract

**Background:**

Multilevel, longitudinal studies are integral to resilience research; however, they are costly and present unique methodological challenges. The objective of this study was to examine the feasibility of study methods (recruitment, retention, data collection) for a large-scale prospective, longitudinal study of resilience among young National Guard recruits.

**Methods:**

This feasibility trial used a pre-test/post-test design with embedded laboratory sub-study. Participants were young military recruits who had recently enlisted in the Army National Guard and had not yet shipped to Basic Combat Training (BCT). Recruitment and baseline data collection (Time 1), which included a battery of computerized self-report measures and neurocognitive tests, were conducted at local armories. Participants completed an online follow-up (Time 2) survey outside of drill training after returning from BCT. A subset of participants was recruited to complete extensive laboratory procedures pre-and post-BCT, including clinical interview, additional self-report measures, and performance on a series of neurobehavioral tasks during electroencephalogram recordings and, at pre-BCT only, magnetic resonances imaging. Feasibility outcomes assessed our ability to recruit, retain, and collect data from participants. Analysis of outcomes was based on descriptive statistics and evaluation of the feasibility of the larger study was based on pre-determined go/no go progression criteria.

**Results:**

All pre-determined progression criteria were met. A total of 102 (97.1%) of eligible military service members consented to participate. Of these, 73 (73.7%) completed the Time 2 survey. Of the 24 participants approached, 14 agreed to participate in the laboratory sub-study, 13 completed follow-up laboratory visits. Overall, completion of online surveys and laboratory tasks was excellent. However, participants had difficulties completing online surveys during BCT and the computerized neurocognitive testing battery at Time 2.

**Conclusions:**

Study methods were feasible, and all predetermined criteria for progression to the large-scale longitudinal study were met. Some minor protocol adaptations were identified from this feasibility study. Lessons learned and recommendations for future research are discussed.

## Introduction

### Background and Rationale

Given the serious hazards inherently associated with the occupation of warfighting, military service provides an ideal context for studying resilience. Numerous longitudinal studies have mapped distinct adjustment patterns (i.e., trajectories) among military service members (MSMs) following combat deployment. While these studies have shown that a resilient trajectory is the most common response following deployment (Bonanno et al., 2012; Polusny et al., 2017; Porter et al., 2017), few prospective studies have investigated trajectories of positive adaptation among young recruits beginning early in their military career (Sefidan et al., 2021). Thus, young military recruits who have recently enlisted and not yet shipped to Basic Combat Training (BCT) provide an especially important population for studying resilience because they have not yet been exposed to military-related stressors, and findings could help guide the development of interventions for young recruits (Polusny & Erbes, 2023).

Although it has become fairly common practice to conduct resilience research with military populations (Galatzer-Levy et al., 2018), most existing studies have relied nearly exclusively on self-report. Although invaluable to research, reliance on self-report measurement alone is associated with a number of limitations, including potential for systematic nonresponse bias and monomethod bias (Doty & Glick, 1998; Semmer et al., 2003). While prospective, longitudinal studies of resilience are beginning to incorporate multilevel approaches (Crane et al., 2012; Schmidt et al., 2015), including neuroimaging data (Roeckner et al., 2021), very few have focused on young military recruits at the onset of their careers (Smaliukienė et al., 2022). To unravel mechanisms contributing to resilience in young recruits, we have planned a large-scale prospective, longitudinal investigation with an embedded laboratory sub-study that will incorporate clinical interview, neurocognitive testing data, and DNA sampling.

Because large, prospective, longitudinal studies – such as the study we have planned – are costly and resource-intensive endeavors, it is vital to first determine the feasibility of proposed study methods that have not been previously tested. While our team has a long history of successfully conducting longitudinal studies with National Guard soldiers in the context of military deployment (Erbes et al., 2017; Ferrier-Auerbach et al., 2010a; Polusny et al., 2009, 2017), we are uncertain regarding the feasibility of recruiting and retaining a cohort of younger military recruits (most of whom are age 18). Studies of health and wellbeing in emerging and young adult populations suggest that these age groups may be especially difficult to recruit for longitudinal studies (Lystad et al., 2022). Previous research has also shown that attrition from longitudinal studies is highest among younger adults, especially those with less education (Young et al., 2006). Some researchers theorize that these age groups are difficult to recruit and retain because emerging adulthood is a period of change, instability, and a desire to socially conform which may pose barriers to research participation (Hanna et al., 2014).

In recent years, longitudinal researchers have shifted away from using well established mailed survey methodology and have increasingly adopted electronic survey methods (e.g., collection of self-administered electronic surveys by email) (Millar & Dillman, 2011). While mailed survey methods have historically yielded excellent response rates (~ 65–80%) (Polusny et al., 2017), this method has considerably greater costs (i.e., paper, postage, mailout, and data entry costs). Electronic survey methods have benefits in terms of convenience, accessibility, and reduced need for staffing and data entry. However, the use of online platforms (i.e., Qualtrics) is not without challenges. Results of meta-analyses indicate that online surveys generally have lower response rates (about 20% lower on the average) than traditional mailed surveys(Daikeler et al., 2020; Shih & Fan, 2009), but some studies suggest that online surveys of young adults may yield higher response rates when participants are initially mailed an invitation to complete an online survey with the option of completing the paper form (Lallukka et al., 2020; Larson et al., 2011).

Few longitudinal studies have incorporated performance-based neurocognitive tests or embedded laboratory methods to evaluate potential mechanisms underlying resilience. Computerized neurocognitive tests, such as the Penn Computerized Neurocognitive Battery (CNB), are now available allowing longitudinal researchers to capture and integrate such data within large longitudinal studies (Moore et al., 2015a). However, the Penn CNB was not originally designed to be administered remotely or on personal devices (Gur et al., 2010), and at the time of this study, test developers recommended that a proctor assist with test administration to troubleshoot any problems the participant may encounter when attempting to access the instrument via their own electronic device. Thus, the feasibility of collecting Penn CNB data from groups of participants in a classroom setting at military installations was unclear, as was the feasibility of participants completing the Penn CNB on their personal devices. While embedded laboratory methods provide valuable opportunities to richly characterize participants, collecting data at laboratory visits places a high burden on participants and can be very costly to conduct. Given requirements of the planned large-scale prospective study to collect extensive laboratory data from participants between baseline assessment and participants’ pending departure for BCT, the feasibility of recruiting participants to take part in the laboratory sub-study as well as collecting complete data in a timely manner was also unclear.

To reduce potential biases associated with reliance on retrospective self-reports, resilience studies ideally collect data characterizing participants’ experience of stressor exposure as close in time as feasible to the actual events. We are aware of few studies conducted by civilian researchers in which data were collected from military personnel while deployed to a combat zone or during military training (Ferrier-Auerbach et al., 2010a). Therefore, we are uncertain about the feasibility of collecting brief survey data from participants during BCT.

### Study Objective

This study aims to assess the feasibility of our study methods in preparation for a future large-scale prospective, longitudinal study. We conducted a 12-month study to evaluate the feasibility of subject recruitment and retention procedures for the longitudinal study and an intensive laboratory sub-study. We pilot tested procedures for collecting neurobehavioral data using web-based surveys that incorporated performance-based neurocognitive testing before and after BCT. We also assessed the viability of obtaining comprehensive neurobiological measurements in a laboratory setting before and after BCT. Furthermore, we explored the feasibility of collecting stressor exposure data during participants’ time at BCT and examined the availability of administrative data for study participants.

## Methods

### Study Design

This feasibility study used a pre-test/post-test design with embedded laboratory sub-study and nested randomized controlled trial (RCT). The pre-test/post-test design will allow us to identify best methods for recruitment and retention with our novel population of young military recruits at the onset of their service. The embedded laboratory sub-study will allow us to test the feasibility of recruiting and retaining young military subjects to participate in time-intensive, in-person study visits during times of transition (before shipping to BCT and immediately upon return from BCT). As a secondary aim, the nested RCT will allow us to test the ability of participants to complete brief online surveys while stationed at BCT.

### Recruitment of participants and planned study timeline for the feasibility trial

This pilot feasibility study was conducted at the Minneapolis VA Health Care System and University of Minnesota-Twin Cities. Inclusion criteria for the feasibility trial were recently enlisted in the Army National Guard, aged 18 or older, and scheduled to ship and return from BCT within the 12-month study period. Those individuals previously exposed to BCT (i.e., as part of previous military service) were excluded.

Participants were recruited at local Army National Guard armories. For a full description, please see the procedure paper of this pilot study (Polusny et al., 2021a). Prior to recruitment briefings, military command provided the research team with a list of potentially eligible participants. In advance of drill training, the study team sent potential participants a letter providing a description of the study and inclusion criteria as well as a brochure containing information about the study. During subsequent drill trainings, investigators conducted briefing sessions with groups of recruits to explain the purpose, nature, and risks of the study. Investigators explained that participation in the study was strictly voluntary. We also emphasized that the information gathered from individuals was confidential including from the National Guard. Participants were informed they would receive invites to complete a self-administered electronic survey after returning from BCT as well as potentially several invites during BCT. Military command was not present during these briefings and was not informed whether recruits chose to participate in the study. Following the briefing, recruits who were interested in participating in the study were provided a participant information packet, including a cover letter, a study identification (ID) card, and a “Keep in Touch” contact information form. The cover letter detailed all procedures, risks, and benefits of participation and reiterated the confidential/voluntary nature of the study. Additionally, it provided contact information for the study as well as other resources (e.g., telephone numbers for the National Suicide Hotline and the University of Minnesota Human Research Protection Program). The ID card, branded study logo, included the participant’s name and study ID on the front, and listed study contact information on the back. Keep In Touch forms collected participant information for tracking subjects longitudinally. Participants were asked to provide address, phone numbers, preferred personal email address, weekly availability for laboratory visits, and information for three alternate contacts.

A subsample of enrolled participants was recruited to take part in a laboratory sub-study. For inclusion in the laboratory sub-study, participants needed to be enrolled in the main study and meet safety criteria for an MRI scan (e.g., no metal implants). Participants also needed to have an expected BCT ship-date more than two weeks after their enrollment to ensure enough time to complete schedule/complete a lab visit before BCT. Lab participants were enrolled sequentially until reaching our goal of 14 subjects. For recruitment, we sent out informational letters and then called participants to schedule visits.

### Data Collection

#### Pre-BCT (Time 1) Assessments

At Time 1, participants were asked to complete an online survey that was administered via the University of Minnesota Qualtrics platform using study Chrome Books and Wi-Fi available at armories. The baseline survey included self-report measures of adaptive functioning, internalizing psychopathology, externalizing problems, and individual differences, as well as demographic information (Polusny et al., 2021b). At the end of the Qualtrics survey, a link transferred the participate to an external site where four tests from the Penn Computerized Neurocognitive Battery (CNB; (Moore et al., 2015b)) were administered. The cognitive battery included the Penn Facial Memory Test which measured episodic memory for faces, the Penn Emotion Differentiation Test which measured social cognition through the ability to distinguish between the emotional intensity of faces, the Penn Continuous Performance Test which measured vigilance and visual attention and the Penn Verbal reasoning test which measured language-mediated complex cognitive ability. Baseline data collection took approximately 75 minutes. Per military regulations, participants were not compensated for the baseline survey completed during drill weekends. However, light refreshments (bottled water and granola bar) were provided at Time 1. As a token of appreciation, all participants were sent a thank you card and military challenge coin following completion of the Time 1 survey. This also provided an opportunity to verify/confirm the participants’ home address.

#### Post-BCT (Time 2) Follow-Up Survey

**Figure 1:**
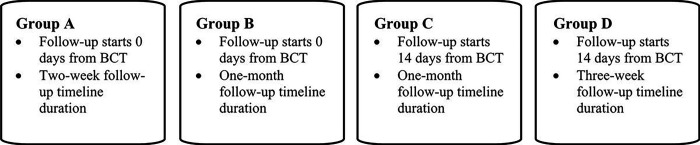
Variations in Timing and Length of Follow-up Procedures

Follow-up data collection using an online Qualtrics survey was conducted outside of drill training after participants returned from BCT. Follow-up outreach procedures used to invite each participant to complete the Time 2 online assessment began by sending a prenotification letter to the participant’s home address and preferred personal email address. Next, participants received an invitation email with a confidential link to the Time 2 survey and a telephone call informing them of the survey. To optimize follow-up survey completion, all survey non-responders received up to 4 emails and 3 calls to remind them to complete the survey. As shown in Fig. 1, we varied the timing (i.e., when outreach procedures started relative to each soldier’s BCT return date) and the length (i.e., number of weeks for follow-up). Initially, we began follow-up outreach procedures by sending prenotification letters on the day the soldier was set to return (day 0) from BCT, followed three days later by the survey email invitation and a two-week follow-up period (Group A). The two-week outreach protocol was too compact to reach participants and receive responses, so the protocol was extended to a month (Group B). The timing of the onset of outreach (e.g., sending letters and initial survey link/call) were the same, but up to 4 reminder emails and 3 calls were administered over 33 days. As we interacted with participants during the follow-up period, we learned that some BCT return dates differed from dates we initially obtained, so some of our initial outreach was occurring before participants had returned from BCT. To account for this, we delayed the start of the entire outreach process by two weeks such that one month follow-up timeline remained the same, but the initial prenotification letter was sent two weeks after participants’ expected return dates (Group C). To explore if it was possible to tighten the length of the follow-up window, we retained the kept the two-week delay in beginning outreach but condensed the reminder emails and phone calls to a three-week span (Group D).

The Time 2 survey included self-report measures administered through Qualtrics and the four tests from the Penn CNB which was linked at the end of the survey. The first version of the invite email contained instructions for the Qualtrics survey, information about digital payment, and detailed instructions for accessing and linking to the CNB using personal devices. If a participant did not complete the CNB at the time they completed the survey, our staff manually sent a separate email containing the CNB link and instructions. Given concerns about the lengthy instructions required to help participants access the CNB, we administered the Time 2 survey and follow-up CNB separately. This allowed us to disentangle estimating the Time 2 survey response rate from that of the CNB.

#### Laboratory Sub-Study

Participants were invited to Minneapolis VA Medical Center to complete a lab visit both prior to shipping and after returning from BCT. Lab participants provided written informed consent prior to completing lab tasks. During both visits, participants completed a clinical interview with a trained researcher. The interview lasted 1–2 hours and included the Clinician-Administered PTSD scale for DSM-5 (CAPS-5; Weathers et al., 2018) and the Structured Clinical Interview for DSM-5 (SCID-5; (First et al., 2016)). Blood for a DNA sample was drawn only at the pre-BCT visit and stored in a specimen freezer at the VA for future analysis. At both lab visits, participants completed a short battery of self-report measures and an electroencephalogram (EEG) assessment. Participants completed the Positive Affect Negative Affect Scale (PANAS;(Watson et al., 1988)) self-report measure of emotional state before starting the EEG assessment. A measure of resting brain activity was administered followed by three EEG tasks. The Dot Probe Task (Frewen et al., 2008; Mogg et al., 2007) measured attentional bias for happy and angry faces to examine effectiveness of executive functions related to attentional control. Participants were presented with a fixation point in the middle of the screen which was followed by two side-by-side faces (one happy or angry and the other neutral). The faces disappeared and behind either the left or right face was a plus sign. Participants were asked to indicate on which side of the screen the plus sign appeared. The Performance Monitoring Task (Gehring & Willoughby, 2002) was a forced choice gambling task with visual feedback that examined neural functioning related to reactivity to environmental stimuli. Participant were told to choose either the left or right box. After blindly selecting, the task revealed if the participant won or lost 5 or 25 points. Finally, the Go/No-Go Task (Gillespie et al., 2022; Lewis et al., 2006) was a task which assessed motor response inhibition and selection with negative feedback for errors. For this task participants were asked to respond for every letter unless the letter was repeated. If they responded to a repeated letter, they saw a red error bar. EEG sessions lasted 2.5–3 hours.

During the Time 1 lab visit, participants were also asked to complete a magnetic resonance imaging (MRI) assessment, which was conducted at University of Minnesota’s Center for Magnetic Resonance Research (CMRR). Data was collected on a 3T Siemens Prisma Scanner with a 32-channel head coil. First, a structural scan was taken while participants remained in a resting state. Next, the previously administered Go/No-Go task was completed along with the Farmer Paradigm (van Meurs et al., 2014). The Farmer Paradigm assessed fear conditioning and fear generalization in the presence of an electric shock through a “virtual farmer” game. Participants must choose between a short road and a long road to get to their crops before wild birds get there first. The short road is more likely to result in getting to the crops. However, on certain trials the stimulus presented means taking the short road will result in a shock, while the long road is always safe. To reduce harm, participants were administered test shocks so they could identify the highest level of shock they were willing to withstand. The MRI session lasted 2.5–3 hours. Participants were compensated $100 for completing the baseline visit to the Minneapolis VA, $100 for completing the baseline visit to CMRR, and $200 dollars for completing their follow up visit.

#### During-BCT Stressor Exposure Surveys

**Figure 2:**
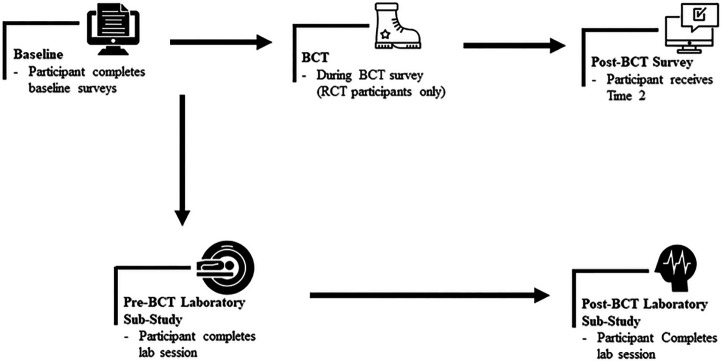
Flow of participant study timeline *Notes:* Basic Combat Training: (BCT). Baseline data collected at National Guard armories; post-BCT survey data collected via Qualtrics surveys sent to participants’ personal email. Pre-BCT lab session consisted of fMRI, EEG, and Clinical Interview session prior to BCT. Post-BCT lab session consists of EEG and Clinical Interview sessions upon return from BCT.

To determine the feasibility of collecting stressor exposure data from participants during BCT, we conducted a small RCT nested within the larger pre-/post-study design. Randomization was assigned by study ID at the time of enrollment using a computer-generated randomization with a simple blocking procedure. A total of 50 participants were set to receive brief online surveys at three timepoints during BCT (Weeks 3, 6, and 9), while the remaining participants did not receive any survey invites during BCT. Each survey contained the 14-item Basic Training Stressor Scale (BTSS), which was estimated to take less than 5 minutes to complete (Polusny et al., 2021b). Two days prior to sending the Week 3 BCT survey invite, we first sent a prenotice email informing the participant they were randomly selected to receive three brief surveys while attending BCT and that they would be receiving an email link to the first survey in 2–3 days. At all three time points, participants randomized to receive surveys during BCT were sent an invitation email containing the survey link, and during next 7 days, non-responders received two reminder emails. Figure 2 displays a progression of the participant recruitment and follow up timeline.

#### Progression and Exploratory Criteria

As part of the UG3 planning phase of our Exploratory/Developmental Phased Award Cooperative Agreement with NCCIH, we established nine predefined quantitative “Go/No Go” progression criteria to determine whether to proceed with the large-scale trial. Table 1 summarizes the feasibility outcomes and progression criteria for each of our study objectives.

#### Feasibility of Study Recruitment Methods

To evaluate our ability to recruit participants into the clinical study, we examined the feasibility of recruitment methods for the survey study and laboratory sub-study. Progression criteria included: enrollment of at least 100 participants, including 25 females, within the 4-month study timeline and a participation rate of at least 75%. Participation rate was calculated as the number enrolled in the trial by the total number military service members approached at recruitment briefing.

Progression criteria for the laboratory sub-study included: recruitment of a minimum of 14 participants to complete lab visits and a recruitment proportion of at least 40%. The laboratory sub-study recruitment proportion was calculated as the number of participants who agreed to participate in the laboratory sub-study by the number of approached by phone.

#### Feasibility of Study Retention Methods

To evaluate our ability to retain participants in the clinical study, we examined the feasibility of response rate for the Time 2 survey and retention rate in the laboratory sub-study. The survey response rate was calculated as the number of participants that responded to a survey by the number of participants initially enrolled in the study. We set the goal of achieving at least 65% response rate to the Time 2 survey. The laboratory sub-study retention rate was calculated as the number of participants who completed post-BCT lab visits by the number eligible for follow up. For the laboratory sub-study, we set the goal of achieving an 85% retention rate. We also had two secondary objectives for analyzing the feasibility of retention methods. We wanted to determine the best schedule of follow up for retaining our sample based on our four different participant outreach protocols. We also examine the response rates of the BTSS.

#### Feasibility of Data Collection Methods

To evaluate our ability to collect study data, we estimated the percentage of participants who provided complete surveys and completed all lab tasks. A complete survey was defined as having less than 10% missing data on key study variables. We set the goal of collecting complete surveys at each timepoint from 90% or more of survey responders. Lab tasks to be completed during a laboratory visit included completion of the clinical interview, self-report measures, four EEG tasks, and at the pre-BCT visit only, the MRI session. We set a goal of achieving a minimum of 80% or greater completion of all lab tasks at each timepoint.

#### Exploratory outcomes

Exploratory outcomes included feasibility of collecting brief surveys during BCT and linking administrative data to participant IDs. Although no progression criteria were defined for these exploratory outcomes, we examined response rates to the brief surveys administered at Weeks 3, 6, and 9 of BCT. To evaluate the feasibility of obtaining administrative data to be used to describe the sample and examine potential response bias, we calculated the proportion of individuals approached to participate in the study, for whom, we could link key administrative data.

#### Sample Size

No sample size calculation was performed for this pilot feasibility study. The sample size was chosen to allow demonstration of the feasibility of study procedures instead of achieving statistical power.

#### Analytical Methods

Feasibility outcomes were calculated as described above (see Table 1 for a full list of study outcomes). Descriptive statistics were used to evaluate the representativeness of the enrolled sample and completeness of the baseline and follow-up surveys. We explored potential response bias for survey non-responders by calculating either the Chi-Square test of independence or using logistic regression analyses. Statistical analyses were conducted using R version 4.1.1.

#### Ethics

This study was approved by the Minneapolis VA Medical Center IRB, the University of Minnesota IRB, and the Minnesota National Guard. Although the study was approved by National Guard command, the research procedures were conducted by a civilian research team with no affiliation to the National Guard.

## Results

### Feasibility of Study Recruitment Methods

Recruitment was completed between December 2017 and February 2018. During this 3-month period, 105 soldiers were approached (1 participant was found to be ineligible for study enrollment due to being age 17 and 1 participant was excluded due to prior BCT exposure) and invited to participate in the clinical study. Among those approached, a total of 101 people, including 31 females, consented to participate and responded to the baseline assessments (participation rate = 97.1%). Table 2 presents demographic and military characteristics of the enrolled sample.

To evaluate our ability to recruit participants into the laboratory sub-study, we targeted the first 24 participants who had ship dates later than two weeks post enrollment. Of the 24 participants we sequentially attempted to contact, we successfully reached 20 out of 24 people. who were screened (all met screening criteria) and invited to participate in the laboratory sub-study. Of those eligible, a total of 16 participants agreed and were scheduled for a lab visit (14 completed pre-BCT lab visits and two cancelled), reflecting a recruitment rate of 58.3%).

### Feasibility of Retention Methods

Of the 101 participants enrolled in the study, four participants did not ship to BCT during the study period and were ineligible for follow-up. A total 97 participants were eligible for follow up, of which, 73 responded to the Time 2 follow-up survey (75.3% response rate).

Of 14 participants recruited for the laboratory sub-study, one did not ship to BCT and was ineligible for follow up. Following BCT, 13 of the 13 eligible participants completed post-BCT follow up laboratory visits (92.9% retention rate).

To further explore our ability to retain subjects in the clinical study, we examined Time 2 survey response rates across four groups of participants with varying follow-up outreach protocols. Response rates did not differ statistically by outreach protocol changes. However, a Chi Square test of independence did indicate that there were significant differences in response rates for the Penn CBN protocol *X*^*2*^ (1, *N* = 97) = 4.40, *p* = .04 (see Table 3).

To test for potential response bias and examine the representativeness of the follow-up sample, we compared Time 2 responders and non-responders on participants’ self-report and neurocognitive test performance collected at Time 1. There was very little response bias between responders and non-responders. We found there was no significant difference in response rates based on gender, race, number of adverse childhood experiences, age at enrollment, or AFQT total scores. However, utilizing a Chi Square Test of Independence we found that proportion of responders differed by education level where participants were split into completed post-secondary degrees, incomplete post-secondary, and no post-secondary education. There was a significant difference in the proportion of responders’ education level *X*^*2*^ (2, *N* = 102) = 8.94, *p* = .01.

### Feasibility of Data Collection

At Time 1, 99 out of 101 participants (98.0%) provided complete surveys (defined as responding to a minimum 90% of key variables). Two participants were not able to complete the CNB because of time restraints. At Time 2, 34 of the 73 people (46.6% completion rate) provided complete surveys when the Penn CNB was included. When we excluded Penn CNB 70 of the 73 participants (95.9% completion rate) provided complete assessments.

For the laboratory sub-study, 13 of 14 (92.9% completion rate) had complete lab visits at the pre-BCT timepoint based on our preset criteria. One participant was unable to complete the MRI because of their profession as a machinist. This was not initially discovered during screening for eligibility for the laboratory sub-study. At lab follow up 13 of 13 participants completed all tasks for a 100% completion rate.

### Exploratory Aims

The nested RCT examined our ability to collect ecologically valid stressor exposure data from participants in the study. Of the 50 participants randomly assigned to receive web-based survey while at BCT, 1 completed the 3-week survey, 2 completed the 6-week survey and 3 completed the 9-week survey. An average of 9.2 emails were sent to the during BCT group.

We found high data linkage rates between our participants and the National Guard provided administrative data (see Table 5 for full results). Rates were calculated based on the 105 soldiers approached. Across the seven key variables, 95 individuals (90%) had no missing data in any variable.

## Discussion

In this pilot study, we aimed to investigate the feasibility of recruiting and retaining young National Guard soldiers in preparation for a multilevel longitudinal cohort study focused on resilience in military populations. We met all pre-set progression criteria examining the feasibility of study methods for the large-scale planned study. Completeness of lab measures also met our progression criteria. Results of the RCT demonstrated that it was not feasible to collect self-reports of BCT stressor exposure during training. Our study demonstrates the adaptability of existing resilience research models to incorporate pre-stressor baselines within a more sophisticated design involving multiple levels of measurement. Importantly, we have successfully established the feasibility of a multilevel approach with an embedded laboratory sub study.

In this study, one of our primary study goals was to examine the feasibility of recruiting a unique population of young adults at the onset of their military careers. This presented us with novel challenges as most existing literature focuses on deployment. Study staff had concerns about participant perceived salience of the study. Much of the current research has been done on soldiers later in their military career who have encountered military stressors and interacted with the concept of resilience more. We thought it would be possible new recruits would not be invested in a study about resilience. However, our initial participation and response rates indicate that our study engagement procedures were effective. We also had concerns about participant engagement due to their assignment to various roles following the uniform BCT experience. Plus, recruits were at varying stages of young adulthood, as some were in high school, and some were older while also juggling the dual responsibilities of National Guard and civilian life. While there were some observed response differences based on education levels, overall, survey response rates were high.

During the randomized trial of collecting self-reports of stressor exposure from participants during BCT, we encountered very low response rates during BCT. This led us to realize that our participants were not realistically available for study participation during this time. Recruits only had limited time, typically 30–60 minutes per week, to use personal electronic devices for leisure activities and communication with family. Responding to survey questions may not be a priority for them during this time. This insight has implications for studies involving similar military populations, including recruitment for personnel in in-theater combat operations, where success of survey administration may depend on survey designs that integrate well into the actual availability during deployment (Ferrier-Auerbach et al., 2010b). In addition, we were initially concerned that persistent outreach during BCT would reduce rapport and subsequent response rates to the Time 2 online assessment. While it is acceptable to briefly reach out by email during military trainings and operations, it may not be realistic to expect high response rates during these events without special accommodations.

Initially, we encountered challenges with achieving acceptable Time 2 online assessment response rates. Our primary objective was to ensure that the follow-up survey occurred as close as possible to the actual stressor probe/challenge event, as we believed it was important for capturing resilience as a dynamic process unfolding over time. However, the post-return period for recruits was quite busy. Many participants were engaged in activities such as moving, starting new jobs, reintegrating into school, etcetera. We also lacked exact return dates. We learned we were not able to obtain precise return dates due to military regulations. Ship dates also frequently shifted slightly before BCT where participants were sent to training early or late. Because there was no communication from study staff planned for the period between study enrollment and BCT, we had no way of knowing if ship dates had shifted, which contributed to the variability of return dates. Consequently, we adjusted our participant outreach protocol by widening the response window to account for availability considerations and shifting the response window later in time to accommodate shifting return dates. After making these modifications, we observed a positive response rate trend. Additionally, participants who received the CNB at the same time as the survey were far less likely to respond during their intended follow-up period. It is possible that this was due to the original email instructions being very long which potentially overwhelmed or confused participants.

At Time 1, we were able to achieve our data completeness goal of 90% for both the online survey and the CNB. Only two participants were not able to complete the CNB due to time constraints. However, we faced challenges with National Guard Wi-Fi, which did not affect assessment completion but caused additional staff burden and reduced standardization of data collection procedures. In addition, we observed that participants became more distracted towards the end of the assessment time, which may have affected data quality. At Time 2, we were able to achieve our completion milestone for the survey portion but not the CNB. We found that participants would fill out the self-report survey but stopped before the CNB when the two were combined in a single link. Despite splitting the CNB into a separate email to make it more accessible, our completion rates did not improve. One possible reason was the length of the combined assessment (cite about survey length and response rates). Additionally, the CNB required specific hardware and software specifications, which created barriers to response. For example, participants were able to fill out the self-report survey on mobile devices, but the CNB required a desktop computer or laptop. The CNB could also only be administered on the Firefox browser with the Adobe Flash plug-in for Firefox. The hardware/software requirements were difficult to trouble shoot remotely and made the CNB inaccessible for many participants.

### Recommendations

Although our study met the progression criteria, we identified areas in our protocol that require improvement for our planned large-scale study and similar studies. We found evidence of response bias among the education groups. In the upcoming main phase (UH3) of our study, we acknowledge the necessity of exerting additional efforts to engage individuals with lower levels of education in our cohorts, as they are at a higher risk of non-response in subsequent survey waves. The RCT of during BCT surveys revealed that soldiers were less likely to respond during training, prompting us to shift our focus towards outreach efforts targeting recruits upon their estimated return. Moreover, we observed that including the CNB assessment in the survey responses led to incomplete data, whereas excluding it allowed us to meet our completeness milestones for the online assessment. Consequently, we will no longer administer the Penn CNB after the participants’ return from BCT. Our results suggest sending separate instructions for the CNB has shown promise in increasing self-report survey response rates. These findings underscore the significance of thoughtfully designing assessment protocols to enhance response rates and data quality in longitudinal studies. Additionally, we recognize the need for concise participant communication in future endeavors.

## Conclusion

The present study achieved its primary goals of investigating the feasibility of recruiting and retaining young National Guard soldiers to participate in a prospective, pre-test/post-test design with embedded laboratory sub-study study on resilience in military populations. Our findings demonstrate that adapting previous research models of resilience to include pre-stressor baselines and multiple levels of measurement was possible, and the approach proved feasible for this unique population of young adults at the beginning of their military careers.

However, several challenges were encountered, including low response rates during BCT and difficulties in achieving completion of the Time 2 CNB assessment. These challenges highlight the need for potential procedural changes in future iterations of the study. Proactive adjustments should be considered to address the issues surrounding participant response and completion rates.

Furthermore, the present study emphasizes the importance of carefully designing assessment protocols to minimize barriers to response, particularly among populations with limited access to technology or subject to external factors that may affect their availability for study participation.

Based on our findings, we conclude that conducting a large-scale longitudinal cohort study on resilience among young National Guard recruits would be feasible with minimal changes to the study protocol. This research would significantly contribute to our understanding of resilience within this specific population.

## Figures and Tables

**Table 1:** Summary of Feasibility Trial Objectives, Quantitative Indicators, and Go/No Go Progression Criteria

Objective	Indicator	Progression Criteria	
1. Demonstrate ability to recruit subjects in the clinical study	Feasibility of subject recruitment (recruitment % for survey study)	Go	Achieve target enrollment of ≥ 100 participants (minimum of 25 females) for inclusion in the pilot survey study
		No Go	< 100 participants enrolled
		No Go	< 25 females enrolled
		Go	Recruitment will require approaching < 133 service members (i.e., > 75% participation rate) within a 4-month recruitment period
		No Go	> 133 service members approached
		No Go	Recruitment requires > 4 months
	Feasibility of subject recruitment (recruitment % for laboratory sub-study)	Go	Recruit at least 14 sample participants for inclusion in the laboratory sub-study
		No Go	< 15 participants recruited to participate in laboratory sub-study
		Go	Recruitment will require approaching < 35 service members (i.e. > 40% participation rate)
		No Go	> 33 participants approached
2. Demonstrate ability to retain subjects in the clinical study	Feasibility of subject retention (survey response rate)	Go	Achieve post-BCT survey response rate (retention rate) of ≥ 65%
		No Go	Post-BCT survey response < 65%
	Feasibility of subject retention (laboratory retention rate)	Go	Achieve post-BCT laboratory visit retention rate of 85%
		No Go	Post-BCT laboratory retention rate < 85%
3. Demonstrate the ability to collect study data	Feasibility of data collection (% survey completion)	Go	Achieve ≥ 90% complete surveys among survey responders
		No Go	< 90% complete surveys
	Feasibility/acceptability of data collection (% laboratory task completion)	Go	Achieve a minimum of 80% or greater completion of all lab tasks
		No Go	
4. Exploratory	Feasibility of survey data collection during BCT		
	Feasibility of linking administrative data		

**Table 2:** Baseline characteristics of the enrolled sample compared to Time 2 survey responders and non-responders

Baseline Characteristic	Time 2 survey responders (n=73)		Time 2 survey non-responders (n=24)		Baseline sample (n = 101)	
	n	%	n	%	n	%
Gender						
Male	51	69.9%	16	66.6%	70	69.3%
Female	22	30.1%	8	33.3%	31	30.7%
Age, years (Mean/SD)	20.8 (3.7)		19.5 (1.9)		20.6 (3.5)	
18 years	19	26.0%	9	37.5%	29	28.7%
19 years	23	31.5%	8	33.3%	31	30.7%
20–24 years	20	27.4%	6	25.0%	28	27.7%
≥ 25 years	11	15.1%	1	4.2%	13	12.9%
Highest Education Level						
No Post-secondary Education	28	38.4%	16	64.0%	45	44.6%
Some Post-secondary Education	29	39.7%	7	29.1.0%	38	37.6%
Completed Post-secondary Degree	16	21.9%	1	4.2%	18	17.8%
Race						
White	56	76.7%	18	75.0%	76	75.2%
Non-White	17	23.3%	6	25.0%	25	24.8%
AFQT (Mean/SD)	67.1 (23.3)		61.3 (21.1)		65.9 (22.6)	

Note. AFQT = Armed Forces Qualification Test

**Table 3:** Comparison of retention outcomes obtained for varying Implementation of Penn CNB

Variable	Penn CNB attached to online survey (n = 55)	Penn CNB separated from online survey (n=42)
Response Rate, n (%)	38 (69.1%)	34 (81.0%)
Number of days to respond from invite date, mean (SD)	12.5 (19.2)	5.7 (8.0)
Minimum number of days to respond from invite date	0	0

Note. **Penn CNB Attached** represents the group that received Time 2 assessments with the Penn CNB attached to the self-report survey and **Penn CNB Separate** is the group that received separate email with Penn CNB link and instruction after completion of the self-report survey.

**Table 5.** Administrative Military Data Linked to Individual Participant Subject Data

Variable	Data provided (N)	% of individuals approached for recruitment with linked data
Graduated BCT	105	100%
Enlistment Date	104	99%
Education	105	100%
AFQT	103	98%
BCT Ship Date	96	91%
Enlistment Type	104	99%
Military Occupation Specialty	104	99%
No missing data for above variables	95	90%

Note. BCT = Basic Combat Training; AFQT = Armed Forces Qualification Test.

## Data Availability

The datasets used and/or analyzed during the current study are available from the corresponding author on reasonable request.

## Key Messages Regarding Feasibility

- *What were the uncertainties regarding feasibility?* The uncertainties regarding feasibility were the ability to recruit, retain, and collect data from young military recruits.
- *What are the key feasibility findings?* All pre-determined progression criteria were met, and the overall trial design and study methods proved to be feasible.
- *What are the implications of the feasibility findings for the design of the main study?* The findings indicate that a full-scale longitudinal study is feasible; however, collecting online survey data from participants while they are at BCT and collecting computerized cognitive measures at follow-up is not.

## Abbreviations

MSM: Military service members
BCT: Basic Combat Training
CNB: Penn Computerized Neurocognitive Battery
RCT: Randomized control trial
BTSS: Basic Training Stressor Scale
EEG: Electroencephalogram
fMRI: Functional Magnetic Resonance Imaging
